# It Is Just a Blood Patch: Considerations for Patients with Preexisting Intracranial Hypertension

**DOI:** 10.1155/2020/8365296

**Published:** 2020-11-10

**Authors:** Devina Shiwlochan, Sargis Ohanyan, Kanishka Rajput

**Affiliations:** Department of Anesthesiology and Pain Medicine, Yale University School of Medicine, 333 Cedar Street, New Haven, CT-06511, USA

## Abstract

Epidural blood patches are routine procedures interventional pain physicians perform for postdural puncture headaches (PDPH), whether it be due to the inadvertent wet tap from an epidural or a diagnostic lumbar puncture. Typically, these patients are relatively healthy and an epidural is relatively straightforward. However, there are cases complicated by a neurologic history such as benign intracranial hypertension. Here, we present a case of a patient with benign intracranial hypertension (BIH) that suffered a postdural puncture headache after a diagnostic lumbar puncture, with no documented opening pressure, continued on acetazolamide. There have only been a small number of documented cases of BIH complicated by PDPH. We discuss the medical management of BIH, how it can exacerbate a postdural puncture headache, our definitive management with an epidural blood patch, and our concerns of rebound intracranial hypertension. We demonstrate that treatment of PDPH in BIH is best managed with image-guided blood patches, with smaller volume of autologous blood, and at a slower rate.

## 1. Introduction

Interventional pain physicians and anesthesiologists get frequent requests for epidural blood patch for the treatment for postdural puncture headache (PDPH).

Postdural puncture headache (PDPH) is one of the most common complications following a lumbar puncture. Defined as an orthostatic headache occurring within five days of a lumbar puncture due to cerebrospinal fluid (CSF) leakage [1], patients present with an orthostatic frontal and occipital headache with associated symptoms such as nausea, vomiting, scalp paresthesias, and rarely more serious sequelae such as epidural hematoma, cranial nerve palsies including visual disturbances, tinnitus, and hearing loss, with severe cases resulting in grand mall seizure [2, 3]. Pathophysiology of postdural puncture headache appears to an imbalance between CSF leak and CSF production, where the leak is greater than the amount of CSF produced. This results in two subsequent events leading to a positional headache: (1) loss of intracranial support and (2) vasodilation [2].

Incidence of PDPH following a lumbar puncture is influenced by many factors, from needle gauge and needle tip design (up to 50% for Touhy needle after accidental dural puncture and up to 36% for 22-gauge traumatic needle used for neurodiagnostic procedures) to patient positioning and practitioner experience [1, 3].

Treatment of PDPH includes conservative measures such as bed rest, adequate hydration (oral or intravenous), abdominal binder, pharmacological treatments such as IV or PO caffeine, ACTH, DDAVP, and/or invasive procedures such as epidural blood patch, epidural saline, or dextran, or in extreme cases, surgical closure of the dural perforation [2].

In this report, we present an interesting case of a postdural puncture headache in a patient with preexisting intracranial hypertension, for whom an epidural blood patch was requested following a lumbar puncture. We will also discuss considerations to bear in mind regarding the approach, volume of blood to be injected, and postprocedure monitoring.

## 2. Case Report

**Figure 1:**
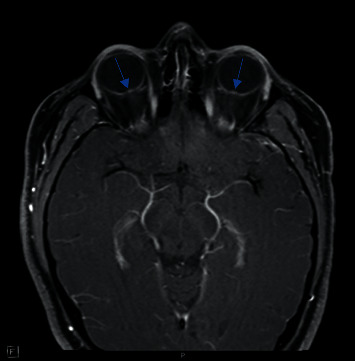
MRI brain. Dilated CSF space is visualised surrounding the optic nerves bilaterally. Possible bilateral optic nerves protrusion into the vitreous space.

**Figure 2:**
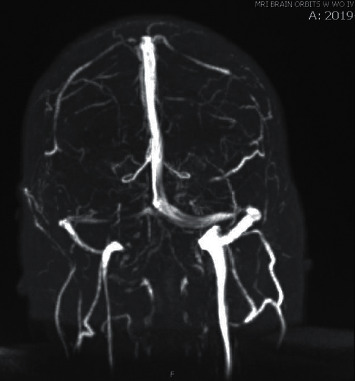
MRV brain. Asymmetric small caliber of the right transverse sinus is noted, possibly suggesting segment at stenosis versus anatomic variant. There is focal stenosis of the distal left transverse sinus near the sigmoid signus.

A 23-year-old female with a history of obesity, cyclical vomiting syndrome, vision changes, and chronic headaches since the age of fourteen, diagnosed as idiopathic intracranial hypertension, was referred to neuro-ophthalmology for the concern of optic disc swelling and papilledema. As per recommendations of the neuro-ophthalmologist, the patient was referred to the Emergency Department (ED) for a magnetic resonance imaging (MRI) of the brain along with a lumbar puncture. MRI showed prominent cerebrospinal fluid (CSF) spaces in bilateral optic nerve sheaths possibly secondary to bilateral stenosis of transverse sagittal sinuses, with normal ventricular CSF (Figures 1 and 2). The patient underwent a lumbar puncture in the ED, with no documented difficulty. Unfortunately, no opening pressure was documented. The patient returned to the ED twice over the course of the next few days, complaining of a positional headache that was “ten times worse,” associated with nausea and vomiting. We were therefore consulted for an epidural blood patch.

The patient did appear to have some clinical features of a PDPH, including an impressive worsening of her headache with position changes, on top of her baseline headache. Interestingly, she also had new radicular pain in the left arm and paresthesias in bilateral legs since the lumbar puncture. No further imaging had been recommended for these new symptoms. In addition, the patient's home dose of acetazolamide was continued for the baseline intracranial hypertension.

Acetazolamide is a carbonic anhydrase inhibitor used in the treatment of chronic benign intracranial hypertension (BIH) and works by reducing CSF production. Continuing the acetazolamide could theoretically be worsening her “low pressure” headache from the dural puncture but could also be helping with her baseline headache and photophobia resulting from the excessive periorbital CSF pressure. The patient had severe photophobia, and hence, a fundoscopic examination had not been repeated. We, therefore, were not sure if performing a blood patch would worsen her intracranial hypertension, especially not knowing what the opening pressure had been at the initial lumbar puncture.

We recommended discontinuing the acetazolamide and trying conservative measures, including intravenous fluid hydration, caffeine, NSAIDs, and bed rest for another 24–48 hours. Her symptoms continued despite the above measures. She therefore underwent an epidural blood patch with Interventional Radiology (due to her body habitus and history of difficult lumbar puncture) at L2-L3 interspace, where a “large” volume of autologous blood of 60 cc (for unclear reasons) was injected. Typical volumes range between 10 and 20 cc of autologous blood. Postoperative symptoms improved markedly, and she was discharged home that same day on acetazolamide.

## 3. Discussion

Treatment of postdural puncture headache depends on its severity. If it is mild and the patient can tolerate an upright position, then a more conservative treatment is implemented, i.e., bedrest, oral/intravenous hydration, and oral analgesics such as caffeine, Tylenol, NSAIDS, and Fioricet [2]. Newer studies have demonstrated benefit with gabapentin, hydrocortisone, serotonin 1d agonists, anticonvulsants, and neostigmine/atropine [4]. Those who fail conservative treatment or are unable to tolerate a sitting up position and perform daily activities, an epidural blood patch is usually the course of treatment. Epidural blood patches provide immediate relief from PDPH, reducing its intensity and duration when compared to conservative treatment [5].

The mechanism by which an epidural blood patch is thought to work is by (1) stopping further CSF loss via forming a clot at the puncture site and (2) creating a pressurelike effect with cephalad displacement of CSF reversing the intracranial hypotension [2]. Performing an epidural blood patch typically involves injecting sterile, fresh autologous blood near the site of previous dural puncture with a volume of around 20 mL, so that there is adequate spread of 4.6 ± 0.9 intervertebral spaces [2].

Epidural blood patch is now considered standard of care to treat acute headaches resulting from a dural puncture. There have been several studies evaluating efficacy of epidural blood patch versus conservative treatment in those with postdural puncture headaches of different etiologies such as lumbar puncture vs epidural. For example, Sandesc et al. showed a statistically significant difference in pain relief on the visual analog scale between the epidural blood patch and conservative measures groups, regardless of the cause of PDPH (*p* < 0.0001). VAS scores were 0.7 ± 0.16 and 7.8 ± 1.2, respectively [6].

In the obstetric population, epidural blood patch is particularly used as the first line of treatment not only because of the efficacy but also because it allows postpartum women to perform their daily activities that require an upright position such as breast feeding and holding their infant—both of which can be greatly inhibited with a PDPH [4]. Epidural blood patches are performed not only for the advantages mentioned previously but also to prevent delayed complications of an ongoing CSF leak such as cranial nerve palsies, seizures, and intracranial bleeding [7].

The decision to perform a blood patch is typically straightforward in a healthy obstetric patient with no prior history of neurologic disease. It becomes more challenging to reach this decision in a patient with preexisting intracranial hypertension. Benign intracranial hypertension is defined as a headache with the following additional criteria: (1) increased CSF pressure without an intracranial mass/lesion/ventricular dilation, (2) normal CSF composition, (3) normal level of consciousness, and (4) normal neurological examination except papilledema and cranial nerve VI palsy. It is a diagnosis of exclusion with the hallmark characteristic of papilledema and normal cranial imaging [8].

Acetazolamide is the first line of treatment, but other management strategies include steroids, repeated lumbar punctures, lumboperitoneal shunting, and optic nerve sheath fenestration.

This interesting case raises several concerns. First, even though the brain MRI showed normal ventricular CSF volume, without the knowledge of an opening pressure at the time of initial lumbar puncture, it is difficult to reach a decision to perform an epidural blood patch as a knee jerk response. If the opening pressure had been documented as low or normal, it could theoretically be safe to treat a potential “low pressure headache” with an epidural blood patch, without overtly worsening the intracranial hypertension. If on the other hand, the opening pressure were documented as high, it would be a relative contraindication to inject more blood in the epidural space due to concerns for a rebound increase in intracranial pressure. In fact, Terson syndrome is a potentially devastating complication related to an intense increase in subarachnoid pressure [9]. Terson syndrome, also known as oculocerebral syndrome of retinal and vitreous hemorrhage [10], has been described with trauma, subdural hematomas, and injection of either saline or blood into the epidural space, which can increase subarachnoid pressure up to 85 cm of water [10]. The resulting extension of the subarachnoid space surrounding the optic nerve and the sudden increase in subarachnoid pressure can result in retinal and vitreous hemorrhage. This increase in pressure can be seen with as little as 15 mL of epidural volume instillation. The first case of Terson syndrome related to epidural blood patch was only recently reported in 2016 [9].

While this outcome is exceedingly rare, anesthesiologists should consider injecting epidural blood slowly and discussion of this complication should be considered in the consent process for patients with preexisting poor vision or concern for elevated intracranial pressure.

How much volume will therefore adequately tamponade the dural puncture without causing a rebound intracranial hypertension and other complications? Although epidural blood patch is one of the most efficacious ways to treat a PDPH with success rates of 85% with first blood patch and 95% success rate with a second one, rare complications including intraventricular hemorrhage and subdural hematoma with as little as 18 ml of blood have been reported. In healthy obstetric patients without BIH, a retrospective study has found no difference in efficacy of blood patch with volumes between 15 ml and 30 ml [11]. It is important to keep in mind that an increase in subarachnoid pressure can be seen with as little as 15 mL of epidural blood volume injection [10]. There is, however, no expert recommendation for an ideal volume in a patient with preexisting BIH. In other words, we did not know how much blood might be enough or too much for this patient. In our case, the patient received 60 cc of blood during her epidural blood patch which could have resulted in the consequences listed above subsequently leading to devastating neurologic injury, longer hospital stay, and financial and long-term lifestyle changes.

There have been just a handful of cases reported, where a patient with a history of benign intracranial hypertension, after failing conservative treatment with acetazolamide, underwent a lumbar puncture which was complicated by PDPH and subsequently underwent an epidural blood patch [8]. The authors, using the blind technique, injected 2–3 ml of blood every 30 seconds for a total of 20 ml of blood into the epidural space. The patient noted immediate resolution of the headache, and the symptomatic headache from benign intracranial hypertension did not return until 1 month later [10, 12].

Our patient did receive a blood patch with a “large” volume of blood in the IR suite and was sent home the same day. It may be reasonable to guide the volume of blood to be injected by the opening pressure at the time of LP. If the patient has a normal opening pressure (possible in this case, since pre-LP MRI showed normal ventricular CSF volume), it may be reasonable to inject the recommended 15–30 ml, with an incremental injection of 2–3 cc every 30 seconds as previously described. In case of high opening pressure, it may be prudent to inject a lower volume at a slower rate so as to prevent a rebound increase in intracranial pressure. Image guidance could theoretically reduce the number of attempts needed to access the epidural space especially if the lumbar puncture procedure was difficult to perform in the first place. The literature suggests that fluoroscopic guidance versus anatomic landmark technique allows for better guidance, a higher success rates, decreased need for repeat blood patches, and decreased incidence of back pain and complications and requires a smaller volume which can be essential in a patient with known intracranial hypertension [13]. A study performed by Al-Hazar found that there was a 95% success rate in epidural blood patches performed under fluoroscopy versus 40% success rate under the traditional methods [13].

Given the rarity of such unique cases, there are no specific guidelines for postblood patch monitoring. Although epidural blood patch helps with rapid resolution of the headache, most experts recommend keeping the patient in the supine position for 30–60 minutes [14]. In an otherwise straightforward situation such as an obstetric patient receiving a blood patch, patients are usually discharged home the same day after monitoring for a few hours and no true guidelines noted. However, the literature suggests that the mass effect of the clotted blood has been reported for up to 7 hours, and it has been proposed that the increase in CSF pressure also may be prolonged [15]. Sperry discusses about a 12 -year-old male who developed severe neurological sequalae after receiving an epidural blood patch for a postdural puncture headache which developed after a lumbar drain placement for a bifrontal craniotomy for resection of a suprasellar pituitary adenoma followed by a transsphenoidal surgery for resection of residual tumor. In this case, autologous blood was administered to the epidural space in slow increments of 0.5 ml/minute, while vital signs and patient response were continuously monitored. The patient received a total of 20 ml of autologous blood and suddenly became bradycardic and unconscious. Follow-up MRI demonstrated significant increase in the size of the cerebral ventricles, including the third ventricle, requiring and external ventricular drain placement [16]. Given this unique case with preexisting intracranial hypertension and no information on opening pressures during the LP with normal intracranial volume appearance on MRI, not to mention the large volume of blood that was injected by IR, our recommendation is to monitor the patient for up to 24 hours after the procedure to monitor for worsening or new neurologic symptoms, which could be attributable to a rebound increase in intracranial pressures along with an early follow-up with the outpatient neuro-ophthalmologist for ongoing management.

## 4. Conclusions

Epidural blood patch is considered standard of care for management of a postdural puncture headache, especially in the obstetric population. The decision to perform a blood patch in a patient with preexisting benign intracranial hypertension should, instead of being a knee jerk response, be one that is more measured and in close consultation with both the neurologist and neuro-ophthalmologist. The volume of blood to be injected may be guided by pre-LP imaging findings as well as opening pressure at the time of LP. Small volumes injected every 30 seconds, using image guidance, could not only improve success rate but also allow an overall smaller volume of injectate. The importance of close monitoring of patients during and for several hours following the procedure, to recognize early signs of a rebound increase in intracranial pressure, cannot be overemphasized.

## Data Availability

No data were used to support this study.
